# Blood mRNA Expression Profiles of Autophagy, Apoptosis, and Hypoxia Markers on Blood Cardioplegia and Custodiol Cardioplegia Groups

**DOI:** 10.21470/1678-9741-2020-0330

**Published:** 2021

**Authors:** Deniz Elcik, Aydın Tuncay, Elif Funda Sener, Serpil Taheri, Reyhan Tahtasakal, Ecmel Mehmetbeyoğlu, Isın Gunes, Omer Naci Emirogullari

**Affiliations:** 1 Department of Cardiology, Erciyes University Medical Faculty, Kayseri, Turkey.; 2 Department of Cardiovascular Surgery, Erciyes University Medical Faculty, Kayseri, Turkey.; 3 Department of Medical Biology, Erciyes University Medical Faculty, Kayseri, Turkey.; 4 Erciyes University Genome and Stem Cell Center (GENKOK), Kayseri, Turkey.; 5 Department of Anesthesiology and Reanimation, Erciyes University Medical Faculty, Kayseri, Turkey.

**Keywords:** Cardioplegic Solutions, RNA, Messenger, Caspase 9, Aspartic Acid, Heart Arrest,Induced, Cardiac Surgical Procedures, Real-Time Polymerase Chain Reaction

## Abstract

**Introduction:**

Blood cardioplegia (BC) and Custodiol cardioplegia (CC) have been used for a long time in open heart surgery and are highly effective solutions. The most controversial issue among these two is whether there is any difference between them regarding myocardial damage after ischemia surgery. In this study, autophagy, apoptosis, and hypoxia markers were investigated and that way we evaluated the differences between BC and CC patients.

**Methods:**

A total of 30 patients were included in this study, using two different cardioplegic solutions. Three different whole blood samples of the patients were taken from a central vein (preoperatively, immediately postoperatively, and one day after surgery). Total ribonucleic acid was extracted from these samples. Quantitative real-time polymerase chain reaction was performed, and changes in gene expression were determined by the 2-∆∆Ct method of relative quantification.

**Results:**

In the CC group, *Beclin* gene expression level was found to be higher and this difference was statistically significant (*P*=0.0024). Similarly, cysteine-aspartic acid protease (*caspas*e) 9 and hypoxia-inducible factor 1α messenger ribonucleic acid (mRNA) gene expression level increased and were significantly different in the CC group. In the BC group, *Beclin* and microtubule-associated protein light chain 3 expressions were higher in the samples taken one day after surgery. *Caspases* 3 and 8 gene expressions were significantly different in the BC group.

**Conclusion:**

As a result of the analysis performed between the two cardioplegia groups, it has been shown that CC harms the myocardium more than BC at the level of mRNA expression of related markers.

**Table:** 

Abbreviations, acronyms & symbols				
AO	= At the end of cardiopulmonary bypass		HDL-c	= High-density lipoprotein cholesterol
AO-1	= One day after surgery		HIF1-α	= Hypoxia-inducible factor 1α
Atg	= Autophagy-related		IR	= Ischemia-reperfusion
BC	= Blood cardioplegia		LC3	= Microtubule-associated protein light chain 3
BMI	= Body mass index		LDL	= Low-density lipoprotein
BO	= Before aortic cross-clamping		mRNA	= Messenger ribonucleic acid
CABG	= Coronary artery bypass grafting		PCR	= Polymerase chain reaction
CAD	= Coronary artery disease		proBNP	= B-type natriuretic peptide
Caspase	= Cysteine-aspartic acid protease		QRT	= Quantitative real-time
CC	= Custodiol cardioplegia		RNA	= Ribonucleic acid
CK-MB	= Creatine kinase myocardial band		TC	= Total cholesterol
CPB	= Cardiopulmonary bypass		TG	= Triglyceride
HDL	= High-density lipoprotein		WBC	= White blood cell

## INTRODUCTION

One of the main treatment options for coronary artery diseases (CAD) is coronary artery bypass surgery, but myocardial ischemia-reperfusion (IR) injury is inevitable in this treatment. Ischemic injury during surgery is directly related to postoperative patient mortality and morbidity. Myocardial protection desired to be provided during coronary artery surgery depends on the adequate access of cardioplegic solutions used to all regions of the heart. It is still controversial which cardioplegia solution is more effective in myocardial protection during cardiac surgery. Although experimental studies show that blood cardioplegia (BC) is more effective in myocardial protection than crystalloid cardioplegia^[1,2]^, clinical studies do not fully support this result.

Cell death and survival rates are important in hemostasis of tissues, pathogenesis of diseases, and development of organs. Apoptosis is defined as a form of cysteine-aspartic acid protease (caspase)-related genetic control cell death and this has been associated with cardiovascular diseases. Apoptosis, one of the forms of cell death in the body, is activated by stimuli defined as external and internal pathways. The pathway resulting in activation of caspase 9 is the mitochondrial pathway. Caspases 3, 6, and 7 are activated by caspases 8 and 9, which causes the division of basic cellular substrates and apoptotic cell death in the external pathway^[3]^. It has been thought that apoptosis, which causes the death of cardiomyocytes, may be responsible for the development and progression of heart failure^[4]^.

Autophagy is a form of cell death that occurs to eliminate unnecessary cells during tissue and organ development^[4]^. Several autophagy-related (Atg) genes/proteins, including Beclin-1 protein (encoded by *BCN1*, a mammalian homolog of yeast *Atg6* gene) and microtubule-associated protein light chain 3 (LC3, homolog of yeast *Atg8* gene), play important roles and are often considered as potential markers of autophagic activity. Beclin-1 (encoded by *BCN1*) and LC3 (homolog of yeast *Atg8* gene) are major mediators of autophagy. LC3 is an essential component for elongation of autophagosomal membranes and Beclin-1 is involved in the very early stage of autophagosome formation (nucleation phase)^[5]^. Autophagy induced by cardiac IR during acute stress is thought to be cardioprotective^[6]^. Although as much as damaged mitochondria and cytochrome c release are potent triggers of apoptosis, the autophagic removal process is a critical antiapoptotic mechanism^[7]^. Indeed, Garcia et al. have reported impaired cardiac autophagy in patients with postoperative atrial fibrillation^[8]^.

There are conflicting studies showing that the effects of BC are superior to or similar to those of Custodiol cardioplegia (CC) in adult patients. This issue has not been fully clarified in terms of autophagy, hypoxia, and apoptosis in the blood of coronary bypass patients. For this reason, we investigated which group could have better results with messenger ribonucleic acid (mRNA) expressions of autophagy, apoptosis, and hypoxia biomarkers.

## METHODS

### Patients’ Samples

The study was performed with 30 patients who underwent randomized coronary artery bypass grafting (CABG). The patients were equally divided into BC and CC groups. The Erciyes University Medical Faculty Ethics Committee approval was obtained for the study and the 1964 Helsinki Declaration (and its later amendments) or comparable ethical standards were followed. Patients with acute coronary syndrome, emergency CABG, chronic renal failure, previous heart surgery history, infective endocarditis, peripheral vascular diseases, and chronic inflammatory disease met the exclusion criteria. Basal blood levels and echocardiographic evaluation were performed before the procedure. Three different whole blood samples of the patients were taken from a central vein for genetic analysis: prior to starting the cardiopulmonary bypass (CPB), at the end of CPB, and one day after surgery. Written informed consent was obtained from the patients before they participated in the research. This article does not contain any studies with animals performed by any of the authors.

### Anesthesia and Surgery

During anesthesia induction, propofol and fentanyl were administered to all patients. Esmeron, propofol, and fentanyl are used in anesthesia infusion. The surgeries were performed by the same surgeon and perfusion team. Median sternotomy, standard CPB, and moderate hypothermia (28-31ºC) were used. In the BC group, the antegrade method was preferred and after starting with 700 cc, 400 cc return was given with a 20 min interval. Custodiol was administered as a single dose of 30 cc/kg. The surgical procedure included left internal mammary artery for left anterior descending artery and saphenous graft for other vessels. According to preoperative echocardiographic evaluations, patients with valve replacement indication (n=5, 3/2) underwent additional valve replacement for coronary operation.

### Biochemical Parameters

Total cholesterol (TC), high-density lipoprotein cholesterol (HDL-c), low-density lipoprotein (LDL)-cholesterol, triglyceride (TG), aspartate transaminase, and alanine transaminase were assayed with the Beckman Coulter analyzer using its specific kits. The level of LDL-cholesterol was calculated using Fridewald’s formula (LDL = TC - HDL-c - [TG/5]). Normal reference values were as follows: TC, 120 - 200 mg/dL; HDL-cholesterol, 40-60 mg/dL; LDL-cholesterol, 100-130 mg/dL; and TG, 35-150 mg/dL. Blood samples were taken from the patients to evaluate renal function. Normal reference values were taken as 5-25 mg/dL for blood urea nitrogen and 0.6-1.2 mg/dL for creatinine. Three different whole blood samples of the patients were taken from a central vein: prior to starting the CPB, at the end of CPB, and one day after surgery.

### Quantitative Real-Time Polymerase Chain Reaction (QRT-PCR) Analysis for Gene Expression

Total ribonucleic acid (RNA) was extracted from whole blood samples of patients using PureZol (Bio-Rad, Hercules, California, United States of America) according to the manufacturer’s instructions. The RNA concentration was determined by a NanoDrop ND-1000 spectrophotometer (NanoDrop Technologies, Inc., Rockland, Delaware, United States of America). RNA (1 µg) was reverse transcribed by a First Strand cDNA Synthesis kit (Roche Diagnostics GmbH, Mannheim, Germany) according to the manufacturer’s instructions. QRT-PCR assay reactions were performed using LightCycler 480 Probes Master and Primer/Probes (Roche Diagnostics GmbH) in a 20-µl reaction volume. Reactions were run in duplicate and QRT-PCR was performed with the LightCycler 480 II instrument (Roche, Germany). The cycling conditions used were: initial denaturation at 95ºC for 10 min, followed by 45 cycles at 95ºC for 10 sec, 60ºC for 30 sec, and 72ºC for 60 sec. Beta-actin (or *ACTB*) and glyceraldehyde-3-phosphate dehydrogenase (or *GAPDH*) were used as housekeeping genes. The changes in gene expression were determined by the 2^-ΔΔ^Ct method of relative quantification.

### Statistical Analysis

Statistical analyses were conducted using IBM Corp. Released 2012, IBM SPSS Statistics for Windows, Version 21.0, Armonk, NY: IBM Corp. The compatibility of the countable variables with normal distribution was checked by the Kolmogorov-Smirnov test. Non-parametric tests were applied to the variables without normal distribution after logarithmic transformation, and parametric tests to those with a normal distribution. Values not compatible with normal distribution were expressed as mean ± standard deviation (or mean±SD). In order to compare the pre-procedure and post-procedure values, binary *t*-test was used for variables with normal distribution and Wilcoxon’s rank test for those without normal distribution. Relationships between the variables were examined using Pearson’s correlation analysis. *P*<0.05 was considered as statistically significant.

## RESULTS

### Clinical Findings

A total of 30 patients, 15 in the BC group and 15 in the CC group, were included in the study. There was no significant difference between the two groups in baseline characteristics (Table 1). Furthermore, there was no significant difference between the two groups in terms of hemodynamic parameters of cardiac rate, systolic blood pressure, and diastolic blood pressure.

**Table 1:** Patients' baseline characteristics.

	Blood cardioplegia (n=15)	Custodiol cardioplegia (n=15)	*P*-value
Age (years)	62.9±8.6	64.7±7.2	0.452
BMI (kg/m^2^)	22.6±2.4	22.5±2.9	0.558
Hemoglobin (g/dl)	13.6±1.6	13.1±1.3	0.596
WBC (10^3µL)	8.0±2.0	8.1±1.9	0.798
Platelet count (10^3µL)	260±79	270±89	0.547
Creatine (mg/dl)	1.0±0.2	0.8±0.1	0.190
Glucose (mg/dl)	98±23	95±19	0.649
Total cholesterol (mg/dl)	177±38	183±41	0.470
LDL (mg/dl)	107±31	114±35	0.323
HDL mg/dl)	27±9	29±9	0.459
Triglyceride (mg/dl)	147±82	152±47	0.474
Troponin	0.11±0.06	0.13±0.03	0.130
CK-MB	24.09±17.6	25.33±20.2	0.888
ProBNP	806±616	607±554	0.147
Hemodynamic parameters			
Systolic blood pressure	121.8±16.5	123.8±19.1	0.664
Diastolic blood pressure	79.2±11.6	77.7±13.1	0.778
Echocardiography			
Ejection fraction	54.7±5.4	55.7±6.3	0.647
Systolic diameters	3.49±0.56	3.32±0.3	0.310
Diastolic diameters	5.18±0.72	4.99±0.44	0.379

BMI=body mass index; CK-MB=creatine kinase myocardial band; HDL=high-density lipoprotein; LDL=low-density lipoprotein; proBNP=B-type natriuretic peptide; WBC=white blood cell

No significant difference was found between the two groups in terms of the echocardiographic parameters left ventricular ejection fraction, left ventricular end-systolic diameter, and left ventricular end-diastolic diameter (Table 1). Although there was no significant difference in postoperative echocardiography, the ejection fraction was better in the BC group than in the CC group (51.0±1.8 *vs*. 48.1±2.4, respectively; *P*=0.4).

Another important data of our study is the cross-clamping times that will affect ischemia and apoptosis. When the cross-clamping duration of the patients was examined, no significant difference was found between the two groups (119.0±27.9 in the CC group *vs*. 127.0 ±36.2 in the BC group; *P*=0.504).

### Blood mRNA Expression Results

**Fig. 1:**
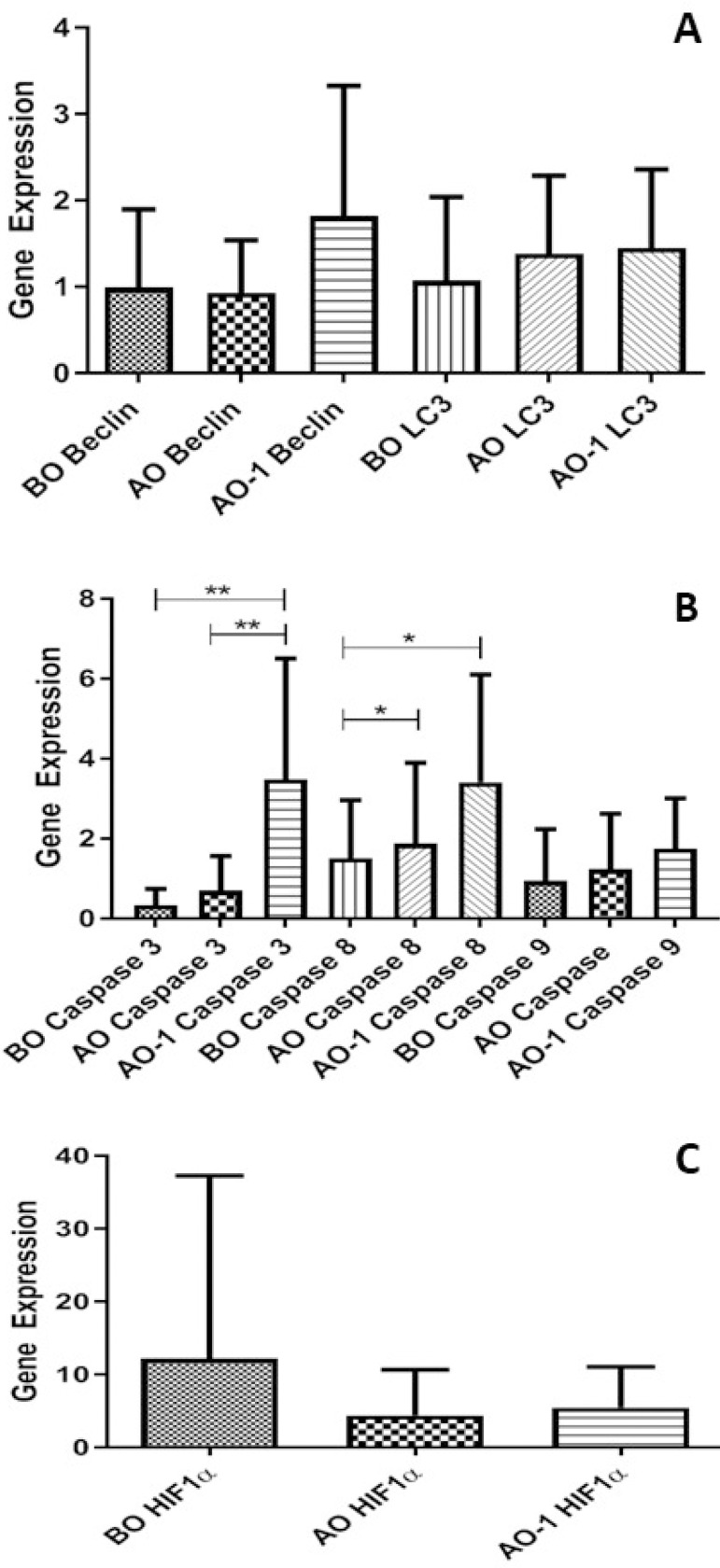
Blood gene expression levels in the blood cardioplegia (BC) group (n=15). A. Microtubule-associated protein light chain 3 (LC3) and Beclin genes as autophagic markers were studied. B. Blood cysteine-aspartic acid protease (caspase) 3, caspase 8, and caspase 9 genes as apoptosis markers were studied (*P<0.05, **P<0.001). C. Hypoxia-inducible factor 1α (HIF-1α) gene as a hypoxia marker was studied in the BC group. AO=at the end of cardiopulmonary bypass; AO-1=one day after surgery; BO=before aortic cross-clamping

Three different blood samples of patients in the BC group were compared among themselves; the *Beclin* gene expression in the blood sample taken on the first postoperative day was found to be increased compared to the *Beclin* gene expression in the blood sample taken before the heart was stopped during surgery and the gene expression in the blood sample taken after the heart was operated, but this increase was not statistically significant. *LC3* gene expression was increased in preoperative blood samples compared to postoperative blood samples, but this increase was not statistically significant (Figure 1A). *Caspase* 3 and *caspase* 8 gene expressions were found to be statistically significant and both of them were increased (*P*<0.001 and *P*=0.0437, respectively). *Caspase* 9 expression did not show a difference between the samples (Figure 1B). There was a statistically significant difference in terms of hypoxia-inducible factor 1α (*HIF-1α*) gene expression (*P*=0.0135) (Figure 1C).

**Fig. 2:**
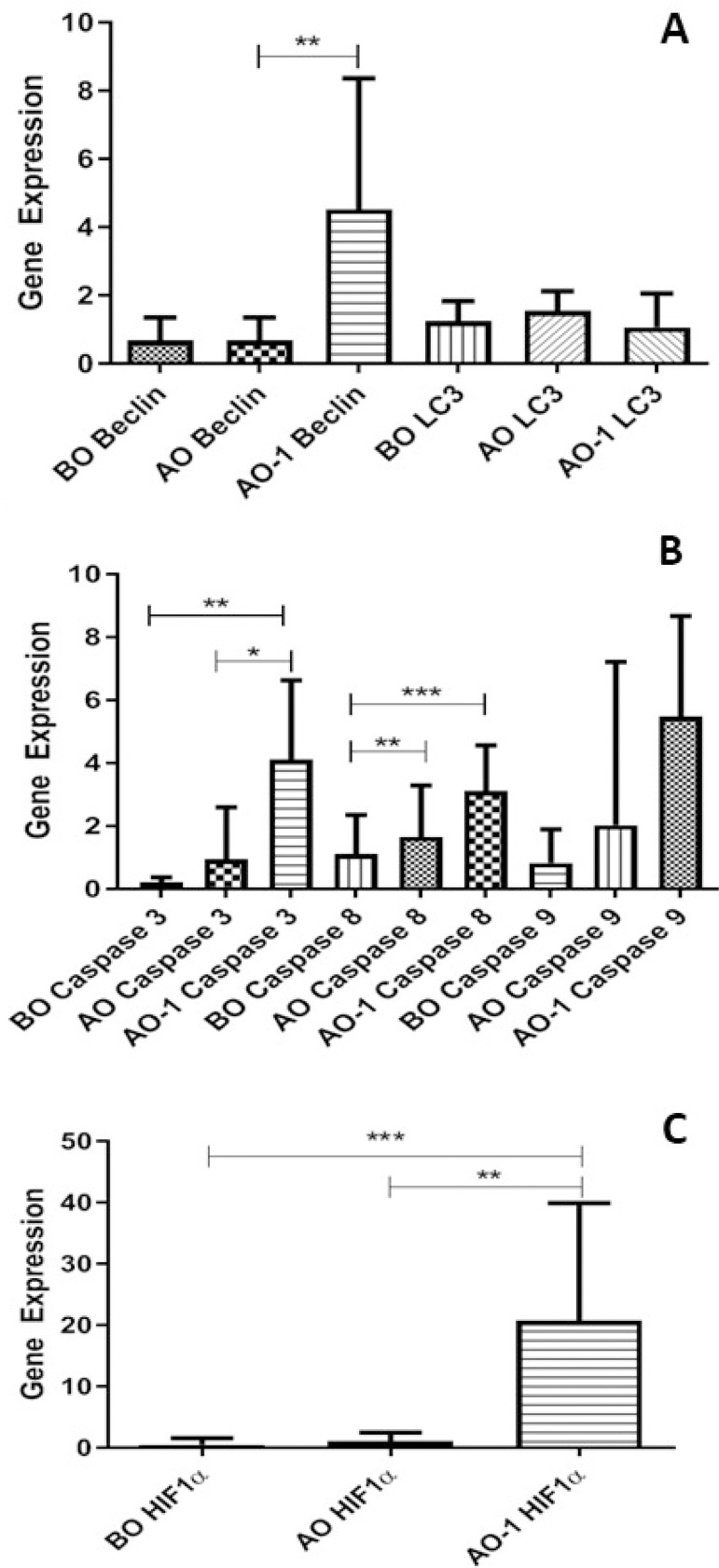
Blood gene expression levels in the Custodiol cardioplegia (CC) group (n=15). A. Microtubule-associated protein light chain 3 (LC3) and Beclin genes as autophagic markers were studied (**P<0.001). B. Blood cysteine-aspartic acid protease (caspase) 3, caspase 8, and caspase 9 genes as apoptosis markers were studied (*P<0.05, **P<0.001, ***P<0.0001). C. Hypoxia-inducible factor 1α (HIF-1α) gene as a hypoxia marker was studied in the CC group (**P<0.001, ***P<0.0001). AO=at the end of cardiopulmonary bypass; AO-1=one day after the surgery; BO=before aortic cross-clamping

Patients in the CC group were compared among themselves; the *Beclin* gene expression in the blood sample taken on the first postoperative day showed a statistically significant decrease compared to the mRNA expression in the blood sample taken preoperatively and in the blood sample taken after the operation (*P*<0.001). No differences were detected in *LC3* gene expression in three different samples (Figure 2A). There was a statistically significant difference in *caspase 3*, *caspase* 8, and *caspase 9* gene expressions (*P*<0.001, *P*=0.0019, and *P*<0.001, respectively) (Figures 2B). There was a statistically significant difference in *HIF-1α* gene expression between the groups (*P*<0.001) (Figure 2C).

When mRNA expression levels of the blood samples taken from the BC and CC patients on the first postoperative day and of the heart samples taken after the operation were compared, Beclin gene expression level was higher in the CC group compared to the BC group and this difference was statistically significant (*P*=0.0024). Similarly, it was found that *caspase 9* and *HIF1-α* gene expression levels were increased and significantly differentiated in the CC group (Table 2).

**Table 2:** Comparison of the six mRNA expression levels between the blood samples taken during the operation and at the first postoperative day of the patients who underwent blood cardioplegia and Custodiol cardioplegia.

Genes	Blood cardioplegia AO (n=15)	Custodiol cardioplegia AO (n=15)	Blood cardioplegia AO-1 (n=15)	Custodiol cardioplegia AO-1 (n=15	*P*-value
Beclin	0.9 (0.5-1.4)	0.6 (0.1-1.1)	1.5 (0.6-2.6)	3.1 (2.3-8)	0.1253/0.0024
LC3	1.4 (0.6-2.1)	1.5 (1.2-1.7)	1.3 (0.5-2.1)	0.8 (0.2-2)	0.3951/0.2208
Caspase 3	0.4 (0.06-1.2)	0.3 (0.1-1.2)	4.5 (2.7-10.)	4.4 (2.4-7)	0.7139/0.3312
Caspase 8	0.9 (0.3-2.6)	1.3 (0.3-3.3)	3.5 (1.1-4.6)	2.5 (2.2-4.1)	0.8302/0.999
Caspase 9	0.5 (0.1-2.2)	0.3 (0.1-1.3)	1.4 (0.9-2.4)	5 (3.2-6.1)	0.1884/0.0001
HIF1-α	0.7 (0.05-4.5)	0.15 (0.1-2.4)	3.8 (0.5-7.2)	9 (5-25)	0.7994/0.0088

AO=at the end of cardiopulmonary bypass; AO-1=one day after surgery; caspase=cysteine-aspartic acid protease; HIF1-α=hypoxia-inducible factor 1α; LC3=microtubule-associated protein light chain 3; mRNA=messenger ribonucleic acid

### DISCUSSION

This study’s results showed that BC provided better protection for myocardial tissue during CABG than CC, as evidenced by the blood expression levels of autophagy, apoptosis, and hypoxia genes. Although myocardial protection methods were not fully affected by hemodynamic, biochemical, and echocardiographic findings, it was concluded from mRNA levels. The prolongation of ischemia during operation and the failure to adequately protect the myocardial tissue affect the results of surgery. The purpose of cardioplegic solutions is to prevent the electrolyte imbalances that may occur during the operation, to protect the myocardial energy, and to increase the durability of myocardium against ischemia and reperfusion by preventing acidosis.

One of the most important factors affecting postoperative mortality and morbidity during cardiac surgery is IR injury. Failure of adequate preservation of the myocardium during surgery, prolonged ischemia duration, and reperfusion injury after ischemia should be prevented; cardioplegic solutions can prevent these by regulating electrolyte balance, preserving myocardial energy, and correcting acidosis that will occur.

BC and CC have demonstrated beneficial effects as measured with the biochemical markers in biological models and patients, although the latter option (intracellular composed solution) may appear more effective^[9]^. BC is used as a standard in myocardial protection during cardiac surgery. However, when BC is used, this should be repeated every 15-20 minutes. At this time, the surgical procedure stops^[10]^. Although this seems to be a disadvantage of BC, it gives very good results in replacing deficiencies. A single dose of solution appears adequate in protecting the myocardium for an extended period. However, clinical studies have not been conclusive regarding which type of cardioplegia provides better protection^[11,12]^. Of course, the biggest disadvantage is that these studies are not homogeneous and have time differences. Our study is more homogeneous in this respect, the difference between the basal characteristic and duration of the forehead as a special group will lead to better interpretation. While more macromolecules are used in the studies^[10-13]^, we have obtained different results by using molecular markers in our study. Differences in electrolyte concentrations between the two cardioplegic solutions may contribute to this finding. Furthermore, the introduction of oxygenated blood into cardiomyocytes, a more effective buffer system, and a better and more uniform capillary flow have been proposed as mechanisms behind better protection with BC^[14]^.

In one study, LC3 gene expression and LC3-II protein levels in peripheral leukocytes were measured in patients with CAD (n=146) and healthy controls (n=87). Both the LC3 gene and LC3 protein expression levels in the peripheral leukocytes were significantly decreased between CAD patients and controls. According to those results, the authors suggested that decreased LC3 gene expression led to decreased autophagosome formation. Therefore, they concluded that autophagy in circulating leucocytes may be involved in the pathogenesis of atherosclerosis and CAD^[15]^. Khalil et al.^[16]^ reported that Atg5 and LC3 genes were significantly down-regulated in the blood samples of 50 cardiovascular patients and 30 healthy controls^[16]^. In our BC group, Beclin and LC3 gene expressions were not significant, but there was a continuous increase. In the CC group, a significant increase was detected between the samples taken at the end of CPB and the samples taken one day after surgery.

HIF-1 is a short-lived heterodimeric receptor that has been shown to respond to hypoxic conditions ubiquitous in the body. HIF-1 adapts the body's metabolism and functions to recover from severe hypoxic conditions including inflammation, sepsis, hypertension, hypervolemic shock, heart or lung disease, and anemia. In these critical situations, HIF-1α dimerizes with the HIF-1β transcription factor to copy various hypoxia-response genes^[17]^. HIF-1α expression was significantly stronger in patients with CAD than controls, and the level of HIF-1α was associated with the severity of atherosclerosis and a higher level of coronary collaterals^[18]^. In our study, HIF-1α expression elevation of the one-day-after-surgery samples comparing to the blood samples taken at the end of CPB showed more hypoxia in the CC group than in the BC group, so we can suggest that BC protects from hypoxia.

Caspase-dependent apoptosis plays a vital role in myocardial apoptosis^[19]^. Caspases are essential in cells for apoptosis, one of the main types of programmed cell death, in development and most stages of adult life. Caspases 2, 8, 9, and 10 trigger apoptosis and are known as upstream or initiator caspases; they activate the executioner or downstream caspases comprising of caspases 3, 6, and 7, which ultimately execute apoptotic cell death^[20]^. In our study, more apoptosis was detected in the CC group; particularly caspase 9 expression was found to be more increased than in the BC group, thus it can be said to have a lower effect on myocardial protection than BC.

### Limitations

The most important limitations of this study are the small number of patients and the fact that this is a single-center study.

## CONCLUSION

As a result of the findings, our study showed that traditional treatment could be better for protecting the myocardium during the surgery by managing the cell death mechanisms. Thus, we believe that these results will shed light on new and larger studies within the near future.

**Table:** 

Authors' roles & responsibilities	
AT	Substantial contributions to the conception or design of the work; or the acquisition, analysis, or interpretation of data for the work; drafting the work or revising it critically for important intellectual content; agreement to be accountable for all aspects of the work in ensuring that questions related to the accuracy or integrity of any part of the work are appropriately investigated and resolved; final approval of the version to be published
DE	Substantial contributions to the conception or design of the work; or the acquisition, analysis, or interpretation of data for the work; drafting the work or revising it critically for important intellectual content; agreement to be accountable for all aspects of the work in ensuring that questions related to the accuracy or integrity of any part of the work are appropriately investigated and resolved; final approval of the version to be published
EFS	Substantial contributions to the conception or design of the work; or the acquisition, analysis, or interpretation of data for the work; drafting the work or revising it critically for important intellectual content; agreement to be accountable for all aspects of the work in ensuring that questions related to the accuracy or integrity of any part of the work are appropriately investigated and resolved; final approval of the version to be published
ST	Drafting the work or revising it critically for important intellectual content; final approval of the version to be published
RT	Drafting the work or revising it critically for important intellectual content; final approval of the version to be published
EM	Drafting the work or revising it critically for important intellectual content; final approval of the version to be published
IG	Drafting the work or revising it critically for important intellectual content; final approval of the version to be published
ONE	Substantial contributions to the conception or design of the work; or the acquisition, analysis, or interpretation of data for the work; final approval of the version to be published
